# Community health worker intervention improves early childhood vaccination rates: results from a propensity-score matching evaluation

**DOI:** 10.1186/s12889-022-14239-w

**Published:** 2022-10-04

**Authors:** Patrick Wightman, Kelly McCue, Samantha Sabo, Rebecca Annorbah, Dulce Jiménez, Vern Pilling, Matthew Butler, Martín F. Celaya, Sara Rumann

**Affiliations:** 1 Center for Population Health Sciences, University of Arizona, Tucson, AZ USA; 2 Center for Health Equity Research, Northern Arizona University, PO Box 4065, 86011 Flagstaff, AZ USA; 3 Center for Biomedical Informatics and Biostatistics, University of Arizona, Tucson, AZ USA; 4 Department of Economics, Brigham Young University, Provo, UT USA; 5 Arizona Department of Health Services, Bureau of Women’s and Children’s Health, Phoenix, AZ USA

**Keywords:** Community health worker, Early childhood, Vaccines, Propensity score matching

## Abstract

**Background:**

Arizona’s Health Start Program is a statewide community health worker (CHW) maternal and child health home visiting intervention. The objective of this study was to test if participation in Health Start during 2006–2016 improved early childhood vaccination completion rates.

**Methods:**

This retrospective study used 11 years of administrative, birth certificate, and immunization records. Propensity score matching was used to identify control groups, based on demographic, socioeconomic, and geographic characteristics. Results are reported by historically disadvantaged subgroups and/or with a history of low vaccine uptake, including Hispanic/Latinx and American Indian children, and children of low socioeconomic status and from rural areas, children with teen mothers and first-born children. The average treatment-on-the-treated (ATT) effect estimated the impact of Health Start on timely completion of seven early childhood vaccine series: diphtheria/tetanus toxoids and acellular/whole-cell pertussis (DTaP/DTP), *Haemophilus influenzae* type b (Hib), hepatitis B (Hep. B), measles-mumps-rubella (MMR), pneumococcal conjugate vaccine (PCV13), poliovirus, and varicella.

**Results:**

Vaccination completion rates (by age five) were 5.0% points higher for Health Start children as a group, and on average 5.0% points higher for several subgroups of mothers: women from rural border counties (ATT 5.8), Hispanic/Latinx women (ATT 4.8), American Indian women (ATT 4.8), women with less than high school education (ATT 5.0), teen mothers (ATT 6.1), and primipara women (ATT 4.5), compared to matched control groups (p-value ≤ 0.05). Time-to-event analyses show Health Start children complete vaccination sooner, with a hazard rate for full vaccination 13% higher than their matches.

**Conclusion:**

A state-operated home visiting intervention with CHWs as the primary interventionist can effectively promote early childhood vaccine completion, which may reduce the incidence of preventable diseases and subsequently improve children’s health. Effects of CHW interventions on vaccination uptake is particularly relevant given the rise in vaccine-preventable diseases in the US and globally.

**Trial registration:**

Approved by the University of Arizona Research Institutional Review Board (Protocol 1701128802), 25 January 2017.

## Background

Reducing and eradicating instances of vaccine-preventable infectious diseases through increased vaccination rates among infants and children remains a mainstay of the US Healthy People initiatives [1] and the WHO Sustainable Development Goals. [2] However, as highlighted by the COVID-19 pandemic, social and structural inequities in vaccine uptake continue, including access to, information about, and perception of the utility and purpose of vaccines. In low-income, minority, and other disadvantaged communities, community health workers (CHWs) are well-positioned to address many of these barriers and increase vaccination rates.

The Centers for Disease Control and Prevention (CDC) recommends seven vaccine series for infants and young children: hepatitis B (Hep. B), diphtheria/tetanus toxoids and acellular/whole-cell pertussis (DTaP/DTP), *Haemophilus influenzae* type b (Hib), measles-mumps-rubella (MMR), pneumococcal conjugate vaccine (PCV13), poliovirus, and varicella. [3] Studies report disparities in vaccine uptake among children associated with markers of disadvantaged socioeconomic status, including poverty status, maternal education, insurance type, and WIC eligibility. [4, 5] Moreover, the association between income and vaccine completion status have increased over time: in 2009, children in low-income households were 9% less likely to complete all seven vaccine series compared to families with household income above $75,000, and by 2018 this difference increased to 37%.[5] The overall early childhood vaccination rates in Arizona reflect the US average, but there are substantial disparities among Medicaid-enrolled children by county and race/ethnicity, and the rate of vaccine exemptions continue to rise. [6].

Social and structural barriers associated with vaccine uptake among disadvantaged and minoritized communities include limited access/availability (e.g. proximity, hours of operation, transportation, fees), inadequate healthcare infrastructure (e.g. scheduling/reminder systems, vaccine storage capabilities), and hesitancy (e.g. misconceptions/fear regarding vaccine safety and purpose and religious or cultural objections). [7–9] Additionally, knowledge gaps in immunization schedules, vaccine contraindications, and control over patients’ immunizations among clinical staff and healthcare professionals may impede providers’ efforts to educate parents on the need to vaccinate their children. [7,10].

CHWs and other similar lay health professionals have effectively helped families overcome many social and structural barriers and have been associated with improved vaccination uptake among infants and children. [11–16] A systematic review of CHW interventions globally included four wherein CHWs encouraged and assisted clients to get vaccinated at clinics via community outreach or home visits. [16] In an evaluation similar to the study presented here, researchers found that US children ‘at risk for poor health outcomes’ who worked with a CHW home visitor in a medical home context had greater up-to-date immunization rates by age two, compared to an inverse-probability-treatment weighted comparison group. [17] This study examines whether the vaccination gap among predominantly Medicaid-enrolled, ethno-racially diverse children in Arizona can be closed by a state-run CHW intervention.

## Methods

### Aim

This study evaluated the effectiveness of a state-run CHW maternal and child health home visiting program in increasing early childhood vaccination uptake. Arizona’s Health Start Program (HSP) is embedded within local and tribal health departments and federally qualified health centers throughout the state to improve maternal and child health outcomes among historically disadvantaged groups, including Hispanic/Latinx and American Indian women, and women of low socioeconomic status. [18,19] Given the heterogeneity of the intervention population, we also examined the impact on several subgroups with either a history of disadvantage or low vaccine uptake.

### Arizona Health Start Program

Initially created in 1984, the HSP has been overseen by the Arizona Department of Health Services’ Bureau of Women’s and Children’s Health since 1992. The program was formalized through state statute in 1994 and has been funded by the Healthy Arizona Initiative lottery funds since 2004. HSP serves 14 communities across the state, “to educate, support and advocate for families at risk by promoting optimal use of community-based family health care services and education services through the use of CHWs who live in and reflect the ethnic, cultural and socioeconomic characteristics of the community they serve.” [5].

HSP is unique in that CHWs are the primary interventionists and home visitors. In 2016, 50 CHWs were employed at HSP, of which 82% identified as white, 62% Hispanic/Latinx, and 16% American Indian. Over half (58%) spoke Spanish and 16% spoke a Native language (Navajo, Hopi, or Apache). Majority (90%) had a high school degree and 10% had a college degree. The caseload of full-time CHWs may reach a total of 40 active clients, who are pregnant or up to two years postpartum. CHWs can meet with their clients up to four times per month through the duration of their enrollment in HSP (e.g. child’s second birthday). The CHWs facilitate prenatal care access and attendance, provide perinatal education, referral services, advocacy to access social services (i.e. food, housing, legal assistance), and emphasize timely childhood immunizations and developmental assessments.

To be eligible to participate in HSP, clients must meet the following criteria: (1) live in a targeted service area, (2) be pregnant or postpartum with a child under age two, and (3) have one or more social or medical risk factors. Social risk factors include marital and residential status (e.g. not married, living with parents or extended family), race/ethnicity (minority), low education level, low income, and insurance status (Medicaid or uninsured). Medical risk factors include presence of one or more chronic diseases, maternal BMI (under- or over-weight), and maternal age (≤ 18 or ≥ 35). [20] In 2016, approximately 51% of clients enrolled during pregnancy, and 49% enrolled postpartum.

### Health Start Program CHW childhood immunization training

Among HSP’s five goals, the program aims to “reduce the incidence of children affected by childhood diseases” and “increase the number of children receiving age-appropriate immunizations by two years of age”. [5] The CHWs are trained to motivate and support their clients through behavior-change activities that promote personal agency and self-efficacy. [19,20] CHWs also receive training from The Arizona Partnership for Immunizations every two years to improve immunization education practices, review state immunization requirements, and learn how to navigate the state immunization registry. CHWs thus support and encourage their HSP clients to complete age-appropriate immunizations for their children by age two. Specifically, during every well-child home visit, CHWs (1) educate families about Arizona school and care facility vaccine requirements, [21] (2) ask families about the child’s immunization status, (3) review and compare the child’s immunization cards to the CDC schedule and the child’s record in the Arizona State Immunization Information System (ASIIS) database (if available), and (4) collaborate with clients’ pediatricians by providing warm-handoffs when available, and assisting with immunization appointment scheduling when needed.

### Study design

This retrospective study evaluates the effectiveness of the HSP intervention by comparing early childhood immunization outcomes among children whose mothers participated in the HSP between 2006 and 2016 to those of a statistically-matched cohort. A detailed description of the methodology can be found in Sabo et al. 2019 and 2021. Briefly, using Arizona birth certificate data, we employed propensity score matching to identify non-participants with the same observable demographic, geographic, and socioeconomic characteristics as the participants. The children of these matched mothers make up our synthetic control group(s). Vaccination information for these children was extracted from ASIIS. The differences in outcomes between the intervention and matched-control groups is often referred to as the average treatment-on-the-treated (ATT) effect, defined as the impact of the program among those who participated.

### Study population

Our study population is comprised of the children of mothers who participated in HSP (intervention) and their matches (controls), as described above. For evaluation purposes, we focus on participants who enrolled in the program prior to the birth of the child, based on enrollment and birth dates. This criterion means that the intervention predates the administration of any immunization, thus the timing of events (enrollment, immunization) supports clear attribution of intervention-control group differences to the HSP intervention. This resulted in an intervention population of 7,218 HSP children, born during 2006–2016.

The population of candidate matches was the approximately 1,000,000 women with a recorded live birth in Arizona during the study period who never enrolled in HSP. Compared to all Arizona mothers who gave birth in this period, HSP mothers were more likely to identify as Hispanic/Latinx (59% vs. 42%) or American Indian (12% vs. 6%), have less than high school degree (33% vs. 22%), and be insured by Medicaid (83% vs. 54%). Given this composition, we also evaluate intervention impacts for the following subgroups: Hispanic/Latinx and American Indian children, children residing in rural border counties, children of teen mothers and those with less than a high school degree, and primipara mothers (Table 4). [18].

### Outcome measures

Our analysis focuses on the seven vaccination series intended for young children, for the following viruses: hepatitis B (HepB), diphtheria and tetanus toxoids and acellular or whole-cell pertussis (DTaP/DTP), *Haemophilus influenzae* type b (Hib), pneumococcal conjugate vaccine (PCV13), poliovirus, measles-mumps-rubella (MMR), and varicella. The CDC-recommended dose schedules for children by age two (plus catch-up time frames) are reproduced in Table 1. [3] We evaluate both completion and timeliness of completion for each series and for all seven series as a group.

**Table 1:**
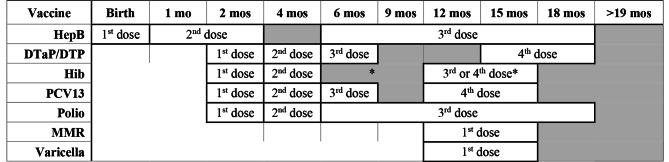
CDC-recommended early childhood immunization schedule, 2021. (Black border: range of recommended ages for all children; Grey boxes: range of recommended ages for catch-up immunization; *Two accepted routine Hib vaccinations: 4-dose series, months 2, 4, 6, 12–15 (ActHIB, Hiberix, Pentacel); 3-dose series, months 2, 4, 12–15 (PedvaxHIB). *Table adapted from*: https://www.cdc.gov/vaccines/schedules/hcp/imz/child-adolescent.html)

### Data sources and variables

Data for this project come from three administrative sources: HSP enrollment records, the Vital Records Birth Database (birth certificates), and ASIIS (immunization records). Using birth certificate data, the matching model included demographic indicators (mother’s age [< 20, 21–25, 26–30, ≥ 30], race/ethnicity, country of origin, marital and cohabiting status); health indicators (presence of pre-pregnancy diabetes and/or hypertension), primiparity (first birth); and socioeconomic indicators (mother’s education [less than high school, high school/GED, some college, 4 or more years of post-secondary education] and primary insurance payer [private/commercial, Medicaid, all others]). Other variables included the birth year, county of residence, and the median household income of mother’s zip code (using indicators for each decile across the distribution). Propensity scores (the likelihood of HSP enrollment) were estimated via logistic regression. This process identified 54,175 appropriate matches for the 7,218 HSP children; for all subsequent comparisons, control group statistics were weighted based on the number of matches and HSP participants within each propensity-score value (designed so to mimic a one-to-one match). Baseline equivalence, the statistical similarity of the intervention and matched-control groups, was verified through t-tests and standardized differences. [18] This matching process was repeated for several distinctive subgroups whose socioeconomic status (e.g. low education) or demographic characteristics (e.g. racial/ethnic minorities) are especially associated with high risk for adverse health outcomes. In each case the matching models are calibrated so that the comparison groups meet the criteria for baseline equivalence prescribed by the What Works Clearinghouse (WWC) Standards Manual (Version 4.1, 2020). [22] Results from these models are available upon request.

In Arizona, in order to access childcare facilities and schools, children aged 18 and younger must be vaccinated and immunizations administered to children must be reported and submitted to ASIIS. [21] The linking of records across all data sources (primarily using names and date of birth) was done through the cooperation of an Honest Data Broker and ASIIS administrators. A limited dataset including HSP enrollment (HSP Database), maternal and child demographics (birth certificates), and vaccination outcomes through 2021 (ASIIS) was then generated for analysis.

Vaccines were identified through ‘Codes for Vaccines Administered’ and ‘Current Procedural Terminology’ codes and descriptions. Age at vaccination was measured by comparing dates of administration and date of birth. Adherence is measured by comparing ages at administration against the CDC-recommended schedules. [3,23] Series are marked as completed if all required doses have been recorded within the appropriate time frames, including during the catch-up period (i.e. older than the recommended ages). Series are marked as completed on-schedule if the final dose was administered within the CDC-recommended age range, conditional on previous doses having happened at appropriate times. In order to standardize the catch-up reporting window across the different cohorts, we report these rates of completion by age five (approximately primary-school age).

## Results

Our summary results for on-schedule completion and completion by age five are presented in Table 2. On-schedule completion is infrequent in this population; however average rates among the HSP cohort as a whole (ATT 2.3% points), children in rural border counties (ATT 4.1), and Hispanic/Latinx children (ATT 4.0) are significantly higher (p-value < 0.05) than their matched comparisons. Among the remaining subgroups none of the differences are statistically significant and American Indians, in fact, do nominally worse (ATT − 1.2) compared to their matches.

**Table 2:** Comparison of completion rates of recommended early childhood immunization series, 2006–2016

	Sample Size (Unweighted)		On-Schedule Completion, All Series				Completion by Age 5, All Series			
HSP Population	HSP	Matches	HSP %	Matches %	ATT	p-value^1^	HSP %	Matches %	ATT	p-value^1^
Statewide	7,218	54,175	25.3	23.0	2.3***	< 0.001	63.8	58.6	5.2***	< 0.001
Rural border counties	2,393	7,093	29.4	25.3	4.1***	< 0.001	71.2	65.1	6.1***	< 0.001
Hispanic/Latinx	4,263	32,719	27.1	23.1	4.0***	< 0.001	67.8	62.3	5.5***	< 0.001
American Indian	852	2,152	24.4	25.6	-1.2	0.574	67.5	62.5	5.0*	0.031
Less than high school	2,364	18,464	21.6	19.6	2.0**	0.094	61.7	56.6	5.1***	< 0.001
Teen (age < 20)	1,264	6,704	23.8	21.7	2.1	0.200	65.5	59.8	5.7**	0.003
Firstborn	3,004	18,266	29.3	27.7	1.6	0.169	67.9	62.8	5.1***	< 0.001

HSP: Health Start Program intervention group; Matches: propensity score matched control group.

^1^ATT p-value based on estimated propensity score.

*** p < 0.001, ** p < 0.01, * p < 0.05

We find stronger treatment effects for full completion by age five. At this point all treatment effects are positive and statistically significant at the 5% level or better, with the exception again of American Indians. The completion rate is nearly 9% higher for the participant group as a whole, and across the subgroups the significant differences range from 7.8% (first-born children, ATT 4.8) to approximately 9.6% (children of teen mothers, ATT 6.1).

Additional detail is reported in Table 3, which shows the on-schedule and by-age-five completion rates for each vaccine series for the HSP children and their matched controls, the percentage-point ATT, and the p-value of the difference. The three 4-dose series (DTaP/DTP, Hib, and PCV13) are largely responsible for the disparity between the rates of on-schedule completion and completion by age five seen in Table 2. Among all HSP children, on-schedule completion rates for these series are 46.5%, 47%, and 54% respectively, where by age five they are 82%, 76% and 74%. We find gains among the matches as well, but the statistically significant treatment effects remain. Late completion driven largely by delays in finishing the 4-dose series is also the pattern among most subgroups of children in this study.

**Table 3:** Early childhood immunization completion by series and timing, 2006–2016

	On-Schedule Completion				Completion by Age 5			
Group & Vaccine	HSP %	Matches %	ATT	p-value	HSP %	Matches %	ATT	p-value
**All Health Start**								
HepB	82.7	77.8	4.98	< 0.001	86.8	82.6	4.25	< 0.001
DTaP/DTP	46.5	43.9	2.66	0.001	81.6	77.6	3.96	< 0.001
Hib	46.8	41.8	5.02	< 0.001	76.0	71.5	4.43	< 0.001
PCV13	53.6	48.0	5.56	< 0.001	73.8	68.6	5.18	< 0.001
Polio	81.4	76.8	4.57	< 0.001	88.2	84.7	3.41	< 0.001
MMR	73.2	68.8	4.38	< 0.001	89.8	86.8	3.02	< 0.001
Varicella	72.2	67.2	5.01	< 0.001	89.4	86.3	3.12	< 0.001
**Rural border counties**								
HepB	88.5	82.9	5.52	< 0.001	91.8	87.3	4.46	< 0.001
DTaP/DTP	52.0	47.5	4.47	0.002	85.2	80.8	4.41	< 0.001
Hib	47.2	43.3	3.85	0.007	80.4	76.1	4.26	< 0.001
PCV13	59.9	53.6	6.29	< 0.001	79.5	74.3	5.14	< 0.001
Polio	87.1	81.3	5.81	< 0.001	92.1	87.5	4.60	< 0.001
MMR	77.6	71.3	6.32	< 0.001	91.5	88.9	2.57	0.003
Varicella	76.4	70.2	6.20	< 0.001	91.3	88.2	3.07	< 0.001
**Hispanic/Latinx**								
HepB	85.5	80.6	4.91	< 0.001	89.2	85.5	3.70	< 0.001
DTaP/DTP	49.1	45.6	3.48	0.001	85.0	81.1	3.96	< 0.001
Hib	47.1	41.4	5.68	< 0.001	78.8	74.9	3.97	< 0.001
PCV13	56.8	51.0	5.84	< 0.001	77.2	72.3	4.92	< 0.001
Polio	84.6	80.1	4.53	< 0.001	90.9	87.4	3.52	< 0.001
MMR	76.6	72.3	4.28	< 0.001	91.7	89.5	2.19	< 0.001
Varicella	75.8	70.8	4.95	< 0.001	91.6	89.1	2.45	< 0.001
**American Indian**								
HepB	85.6	80.7	4.83	0.008	90.0	86.7	3.31	0.033
DTaP/DTP	42.6	42.7	-0.06	0.981	85.7	81.0	4.69	0.009
Hib	58.8	54.7	4.06	0.091	81.2	76.8	4.38	0.026
PCV13	52.7	46.7	6.04	0.013	76.3	71.4	4.92	0.021
Polio	82.9	78.5	4.38	0.022	90.8	88.8	2.04	0.163
MMR	72.9	69.9	3.02	0.168	94.5	92.3	2.23	0.064
Varicella	71.0	68.2	2.81	0.207	94.5	91.5	2.96	0.016
**Less than high school**								
HepB	82.6	77.8	4.82	< 0.001	87.3	83.4	3.84	< 0.001
DTaP/DTP	43.1	38.9	4.24	0.003	81.2	77.2	3.98	< 0.001
Hib	42.9	38.3	4.64	0.001	74.6	70.3	4.31	< 0.001
PCV13	49.7	43.8	5.89	< 0.001	71.5	66.8	4.72	< 0.001
Polio	80.6	76.1	4.53	< 0.001	88.6	85.2	3.35	< 0.001
MMR	71.2	65.9	5.32	< 0.001	89.9	87.1	2.87	0.002
Varicella	70.3	64.1	6.21	< 0.001	89.8	86.3	3.44	< 0.001
**Teen (age < 20)**								
HepB	85.7	79.3	6.43	< 0.001	90.5	84.0	6.55	< 0.001
DTaP/DTP	44.5	41.8	2.67	0.176	83.9	79.7	4.15	0.007
Hib	45.1	40.5	4.57	0.020	76.7	73.5	3.21	0.062
PCV13	53.4	46.9	6.48	0.001	75.0	69.3	5.69	0.001
Polio	82.7	77.8	4.89	0.002	91.2	86.3	4.90	< 0.001
MMR	70.4	66.3	4.12	0.026	91.9	88.0	3.85	0.001
Varicella	69.9	64.6	5.35	0.004	91.5	87.4	4.13	< 0.001
**First born**								
HepB	84.9	80.7	4.20	< 0.001	87.1	83.9	3.18	< 0.001
DTaP/DTP	51.1	50.3	0.80	0.537	82.3	79.8	2.49	0.014
Hib	52.4	48.0	4.41	< 0.001	78.8	75.5	3.34	0.002
PCV13	60.3	54.7	5.59	< 0.001	78.2	73.6	4.63	< 0.001
Polio	84.5	81.4	3.09	0.001	89.0	86.7	2.34	0.005
MMR	76.8	72.9	3.91	< 0.001	89.5	87.6	1.89	0.022
Varicella	75.6	70.9	4.69	< 0.001	89.0	86.9	2.14	0.011

HSP: Health Start Program intervention group; Matches: propensity score matched control group.

*ATT p-value based on estimated propensity score.

The subgroup results largely follow the same patterns we saw in the Table 2 summary measures: HSP is especially effective in the rural border counties, for Hispanic/Latinx children, and children of mothers with less than a high school education. We also find relatively large on-schedule and by-age-five ATTs among children of teen mothers. Among first-born children, HSP is more effective with respect to on-schedule completion.

Finally, Table 4 reports the estimated hazard ratios associated with HSP participation, along with their 95% confidence intervals (CI), from a set of simple Cox proportional hazard models. The event modeled is series completion with time measured as the age of the child in months. These regressions were limited to HSP participants and their matches, with the post-match weight applied to the comparison groups. Among the population as a whole, HSP participation is associated with a 13% higher “hazard”, i.e. these children are fully vaccinated (with respect to these 7 series) sooner. Only among American Indians is this treatment effect not significant and less than 10%. Across subgroups and individual vaccination series the treatment effect averages just under 12%, with especially strong treatment effects again in the rural border counties, and among Hispanic/Latinx children and those whose mothers had not finished high school.

**Table 4:** Time to Completion, Cox Proportional Hazard Models

	HSP	Rural Border Counties	Hispanic/ Latinx	American Indian	Less than High School Education	Teen Mother	Firstborn
All Shots	1.13***	1.18***	1.15***	1.08	1.14***	1.14*	1.12***
	(1.08, 1.18)	(1.10, 1.26)	(1.09, 1.21)	(0.96, 1.21)	(1.05, 1.22)	(1.03, 1.26)	(1.05, 1.19)
HepB	1.11***	1.13***	1.11***	1.09	1.10**	1.17***	1.09**
	(1.08, 1.16)	(1.07, 1.20)	(1.06, 1.16)	(0.98, 1.20)	(1.04, 1.18)	(1.07, 1.27)	(1.03, 1.15)
DTaP/DTP	1.10***	1.15***	1.11***	1.09	1.11**	1.09*	1.05
	(1.06, 1.14)	(1.08, 1.22)	(1.06, 1.17)	(0.99, 1.21)	(1.04, 1.18)	(1.00, 1.19)	(0.99, 1.11)
Hib	1.14***	1.12***	1.13***	1.13*	1.14***	1.11*	1.11***
	(1.09, 1.18)	(1.05, 1.19)	(1.08, 1.19)	(1.02, 1.26)	(1.07, 1.22)	(1.01, 1.21)	(1.04, 1.17)
PCV13	1.14***	1.15***	1.14***	1.15*	1.15***	1.16**	1.13***
	(1.10, 1.18)	(1.08, 1.22)	(1.08, 1.19)	(1.03, 1.29)	(1.07, 1.23)	(1.06, 1.27)	(1.06, 1.19)
Polio	1.10***	1.14***	1.12***	1.08	1.11**	1.13**	1.07*
	(1.07, 1.14)	(1.08, 1.21)	(1.07, 1.17)	(0.97, 1.19)	(1.04, 1.18)	(1.04, 1.23)	(1.01, 1.13)
MMR	1.10***	1.12***	1.09***	1.11*	1.12***	1.13**	1.07*
	(1.06, 1.14)	(1.06, 1.19)	(1.05, 1.14)	(1.00, 1.22)	(1.05, 1.19)	(1.04, 1.23)	(1.01, 1.13)
Varicella	1.11***	1.13***	1.11***	1.13*	1.15***	1.16***	1.09**
	(1.08, 1.15)	(1.06, 1.20)	(1.06, 1.16)	(1.02, 1.24)	(1.08, 1.22)	(1.07, 1.26)	(1.03, 1.15)

Hazard ratios associated with HSP participation (intervention) indictor, 95% CIs reported in parentheses. Estimation population is limited to HSP participants and matches, and weighted using weight based on number of matches and intervention persons within propensity score value.

* p < 0.05; ** p < 0.01; *** p < 0.001.

### Sensitivity analysis

WWC standards for establishing baseline equivalence in propensity score matching analyses are based on standardized (effect size) differences in variables used to identify matches between intervention and matched-control groups. In groups where all differences fall below 0.05, no further adjustments are needed. Where differences are between 0.05 and 0.25, guidelines suggest additional adjustments, including regression adjustments. While the standardized differences between the HSP group as a whole and their matches (reported in Table 5) are all below the 0.05 threshold, for many of the subgroups (not reported, available upon request), even with additional calibration, a small number of differences fall in the middle range. Accordingly we applied additional regression adjustments: in the form of logit models for the dichotomous outcomes, and additional Cox proportional hazard models for the time-to-completion outcomes. Again, per WWC guidelines these models include the HSP treatment indicator, all variables used in the matching models, and the matching-derived weights described earlier. In no case were the results reported here substantially different, either with respect to the magnitude of the treatment effects or their statistical significance.

**Table 5:** Baseline matching results for Health Start Program intervention and matched control groups compared to state, 2006–2016

	All AZ Births %	HSP %	Matches %	p-value	Standardized Difference
**N (Unweighted)**	966,809	7,212	53,948		
**Maternal age**					
Age < 20	9.9	17.5	16.7	0.216	0.02
Age 20–24	25.3	34.4	34.8	0.624	
Age 25–30 (ref)	34.0	28.4	28.5	0.985	
Age > 30	30.8	19.7	20.0	0.573	
**Maternal race/ethnicity**					
White	42.4	24.0	24.0	1.000	0.02
American Indian	6.0	11.8	11.8	0.938	
Hispanic/Latina	41.8	59.1	59.4	0.635	
Other race/ethnicity (ref)	9.8	5.1	4.7	0.233	
**Maternal nativity**					
Mother born in US (ref)	73.6	68.6	69.2	0.472	0.01
Mother born in Mexico	18.7	27.9	27.4	0.503	
Mother born outside US	7.6	3.5	3.4	0.856	
**Maternal education**					
Less than high school/missing (ref)	23.3	33.1	33.7	0.459	0.02
High school/GED	28.8	35.7	35.4	0.728	
Some post-secondary	25.2	23.8	23.2	0.444	
4-year degree or more	22.5	7.4	7.5	0.799	
**Insurance/payer**					
Private/commercial insurance	41.1	13.7	13.4	0.527	0.02
Medicaid	53.8	82.5	83.1	0.343	
Other insurance (ref)	5.1	3.8	3.5	0.451	
**Married**	54.5	37.8	37.8	0.986	0.00
**Cohabiting**	75.6	62.4	62.1	0.744	0.01
**Married & Cohabiting**	52.0	34.7	34.7	1.000	0.00
**First birth**	36.9	41.6	40.8	0.310	0.02
**Maternal pre-existing health risk** ^**1**^	8.3	11.3	10.9	0.427	0.01

HSP: Health Start Program intervention group; Matches: propensity score matched control group.

^1^Presence of pre-existing (non-gestational) diabetes and hypertension.

All models control for median income at the zip code level, county of residence, and year of birth. Participant subgroups matching models may include additional interactions between controls to achieve baseline equivalence. Full tables available upon request.

## Discussion

### Key findings

The majority of US children generally complete recommended vaccines by age two, [1] but significant sociodemographic disparities persist. [24, 4] On-schedule early childhood vaccination completion is important to mitigate vaccine-preventable diseases and achieve herd immunity, particularly during vulnerable years. However, delayed completion of vaccinations, even by age five, is still a notable feat considering the social and structural barriers experienced by many families with young children. This study revealed that completion of all seven CDC-recommended early childhood vaccine series by age five is 8.7% (ATT 4.8) higher for all children enrolled in HSP, compared to a matched-control group, with a 13% higher hazard rate with respect to time to completion.

Several factors impact immunization uptake, such as systems barriers (e.g. vaccine availability, appointment availability, healthcare staff vaccine beliefs/education, language barriers, insurance coverage) and parent/patient barriers (e.g. socioeconomic status, vaccine safety misinformation, ethical/philosophical/medical/religious beliefs, transportation).[4,7–9,25–27] Findings from this study, which showed higher early childhood vaccination completion rates among mothers in rural border counties, Latina and American Indian mothers, mothers with less than high school education, teen mothers, and primipara mothers, are consistent with other international [11–13, 28 and national studies 17,29] which show that community-based CHW programs strengthen child immunization uptake, especially within low income communities. [30] It is well documented that CHWs, through their trusted relationships, have a deep understanding of the community they serve. [31] The rapport CHWs build with clients enable them to communicate complex scientific information in a manner that is culturally relevant and provides critical advocacy combined with individual capacity-building, empowering clients to take action about their health. [27,31].

High rates of child immunizations are associated with a substantial annual cost savings. Typical estimates indicate that $1 spent per child vaccination results in over $10 saved to society (e.g. healthcare/caregiving cost savings, illness/death averted, health/productivity gained, community impact and empowerment). [24,32-35] The Vaccines for Children (VFC) program is specifically tailored to provide the seven vaccines series at no cost to children who are Medicaid eligible, uninsured or under-insured, and/or who identify as American Indian/Alaska Native. [9,24,25] However, the VFC program may place a high burden on pediatricians, who reportedly spend a significant amount of time encouraging parents to vaccinate their children. [36] Thus CHW interventions like the Health Start Program may be effective in not only encouraging and increasing child vaccination rates generally, but also in supporting clinics and physicians through parent and family vaccine education and navigation provided by the CHWs (ideally complementing and amplifying physician efforts). In 2016, HSP served 2,544 unique clients and conducted 16,647 visits, which averages $1,090 per client. As demonstrated by this study, all HSP participants, as a group and in high-risk subgroups, showed higher rates of immunization completion.

With respect to our study population and study period, this is the largest evaluation of a CHW program on early childhood immunization completion in the US to date, and these results constitute valuable evidence of the efficacy of the ability of CHWs to address ethno-racially and geographically diverse, and socioeconomically disadvantaged populations. [37] Although several rigorous studies of prenatal home visitation programs exist, most utilize a combination of licensed health professionals, such as nurses and social workers to achieve outcomes. [38–40] HSP is unique in that it is one of few programs in which CHWs are the sole interventionist and home visitor. [37–39, 41,42] This has important implications for successful programmatic planning, replication, feasibility, sustainability, and implementation. HSP is also unique in that most sites (8 of 14) have access to the ASIIS database, enabling CHWs to view client immunization records in real time. Expansion of ASIIS availability and supplemental training to all HSP sites would increase capacity of CHWs to assist more clients with vaccine compliance. Enabling CHWs in other states to view immunization databases may be a replicable and viable option to increase on-schedule early childhood vaccine completion across the US.

### Limitations

ASIIS is a state-administered registry and so individuals who leave Arizona prior to finishing a given schedule are counted as incomplete. Because CDC early childhood immunization recommendations are given in months, timeliness of vaccine adherence and completion is based on patients’ age in calendar months. Thus, vaccines administered ahead of schedule are considered ‘invalid’. This affects a very small number of children in this study; however, our results may undercount the rate of children with completed immunization series. A more significant limitation (shared by all propensity score matching designs) is the assumption that the observed factors in our matching model explain HSP participation to the extent that the influence of any unobserved factors is negligible. While the presence of social risk factors are priority criteria for HSP eligibility, this information is not available for non-participants (and rarely collected in large surveys or administrative databases), therefore, we are unable to account for these in identifying the control groups. At the same time, if these factors are over-represented in the HSP population relative to their matches, the true program effect may be underestimated. Conversely, participation in HSP is voluntary, thus enrollment itself may be an indicator of characteristics that are also likely associated with increased awareness, vigilance, etc., with respect to their children’s health (i.e. vaccination status). To the extent that this self-selection is unaccounted for in the matching criteria, the true program effect may be overstated.

Finally, Arizona has a comparatively unique population, in part due to its proximity to the Mexico border and its large, sparsely populated rural areas, as well as home to 22 federally recognized American Indian communities. This is reflected in the demographic heterogeneity among HSP participants and, as a result, the present analysis may have limited relevance to other states and populations.

## Conclusion

This study found that families in the CHW home visiting intervention were significantly more likely to report better vaccination outcomes across seven early childhood immunizations, individually and grouped, compared to families who did not participate in the program. These outcomes include uptake and adherence and completion, on-schedule and overall. As demonstrated by HSP, all participants, regardless of income, race, ethnicity, education status, or geographic location benefit from a CHW model that specifically promotes child immunization completion. The global COVID-19 pandemic and the urgency to prevent the rapid spread of SARS-CoV-2 through the universal vaccination of both adults and children brought to light many historical, latent attitudes of vaccine hesitancy in the US, especially among populations at greatest risk for COVID-19 morbidity and mortality. [27] For many minority and disadvantaged communities this reaction is highly contextual, rooted in historical systemic and inequitable social and economic policies. CHWs are especially well-positioned to counter this and other barriers that contribute to disparities and inequities in vaccine uptake. [43, 5,23,24, 33–35,44].

## Data Availability

The datasets used and/or analyzed during the current study are available from the corresponding author on reasonable request.

## Abbreviations

ASIIS: Arizona State Immunization Information System.
ATT: average treatment-on-the-treated.
CDC: Centers for Disease Control and Prevention.
CHW: community health worker.
COVID-19: coronavirus disease 2019.
DTaP/DTP: diphtheria/tetanus toxoids and acellular/whole-cell pertussis.
GED: general education diploma.
Hep. B: hepatitis B.
Hib: *Haemophilus influenzae* type b.
HSP: Health Start Program.
MMR: measles-mumps-rubella.
PCV13: pneumococcal conjugate vaccine.
SARS-CoV-2: severe acute respiratory syndrome coronavirus 2.
US: United States.
VFC: Vaccines for Children.
WHO: World Health Organization.
WIC: Women, Infants, and Children.
